# Clinical, radiological, and pathological features of 33 adult unilateral thalamic gliomas

**DOI:** 10.1186/s12957-016-0820-x

**Published:** 2016-03-10

**Authors:** Peng Zhang, Xingchao Wang, Nan Ji, Jian Xie, Jinsong Han, Xiaohui Ren, Guidong Song, Ruofei Wu, Liwei Zhang, Zhixian Gao

**Affiliations:** Department of Neurosurgery, Beijing Tiantan Hospital, Capital Medical University, Beijing, 100050 China; China National Clinical Research Center for Neurological Diseases (NCRC-ND), Beijing, 100050 China; Beijing Key Laboratory of Brain Tumor, Beijing, 100050 China; Department of Neurosurgery, Beijing Puren Hospital, Beijing, 100069 China

**Keywords:** Unilateral thalamic glioma, Prognosis, Gross total resection

## Abstract

**Background:**

Unilateral adult thalamic gliomas are rarely reported. In this study, the authors aimed to analyze the clinical, radiological, and pathological features of adult primary unilateral thalamus gliomas (UTGs).

**Methods:**

Clinical data of 33 UTGs in adults who underwent surgical treatment between 2005 and 2014 at the Beijing Tiantan Hospital were collected and retrospectively studied. Follow-up evaluation was performed.

**Results:**

This study included 21 males and 12 females with a mean age of 43.1 years. The most common symptoms were headache (75.8 %, 25/33 patients) and motor deficits (42.4 %, 14/33 patients). Radiological results showed that enhancement was common (90.9 %, 30/33 patients) and included cystic appearances in 9 cases (27.3 %). All patients underwent maximal safe tumor resection. Gross total resection (GTR) was achieved in 19 cases, subtotal resection (≥80 %) in 9 cases, and partial resection (<80 %) in 5 cases. Molecular pathology results were available in 15 cases. After surgery, 25 patients received postoperative adjuvant therapy based on the remaining pathology. The median follow-up period of all 33 patients with UTGs was 17 months (1 week~49 months). Twenty-four patients experienced tumor recurrence. The 1-year and 2-year progression-free survival (PFS) rates were 49.0 and 10.2 %, respectively. The 1-year and 2-year overall survival (OS) rates were 68.1 and 25.9 %, respectively. Survival analyses revealed that several predictive factors were correlated with better prognosis, among which, GTR and tumor with cystic appearances were significantly associated with a longer survival.

**Conclusions:**

Adult UTGs displayed a wide spectrum of clinical features. GTR can be achieved in adult UTGs with acceptable complications and conferred a better prognosis. Tumor with cystic appearance may indicate better prognosis. More patients and longer follow-up periods are needed to further elucidate the biological features of adult UTGs.

**Electronic supplementary material:**

The online version of this article (doi:10.1186/s12957-016-0820-x) contains supplementary material, which is available to authorized users.

## Background

Thalamic glomas represent 1 to 5 % of brain tumors [1–7]. This disease has been investigated in several studies spanning several decades. However, many previous series have combined both adult and pediatric populations, despite a highly differential prognosis between the two groups [8–16]. Moreover, several previous studies have assessed both tumors arising in the basal ganglia and other diencephalic structures [6, 17–22] and bilateral thalamic gliomas [10, 23–27], despite the varying characteristics of these gliomas and their associated treatment plans.

Due to decades of improvement in imaging modalities and surgical techniques, a clear preoperative anatomical picture and a more aggressive surgical treatment of these deeply seated lesions are now available. Several recent studies have revealed a more acceptable prognosis among pediatric thalamic glioma patients [15, 28, 29]. However, it is uncertain whether a more radical surgical procedure would benefit adult patients, and the prognosis of this age group remains unknown. In this series, we retrospectively reviewed patients with primary unilateral thalamic glioma who underwent surgical operation at our hospital over a period of 9 years. The aims were to clarify the clinical, radiological, and pathological features and the behavior and response to treatment and to identify the prognostic factors of this diverse group of tumors.

## Methods

### Patient characteristics and inclusion/exclusion criteria

Consecutive patients’ notes and imaging studies were reviewed; all included patients underwent operation between 2005 and 2014 at the Beijing Tiantan Hospital. Informed consent was provided by all patients or their family members under an Institutional Review Board-approved protocol at the Beijing Tiantan Hospital of the Capital Medical University.

Our current study used the following inclusion criteria: (1) histological diagnosis of gliomas and (2) patient age older than 18 years at the time of diagnosis while gliomas originating from adjacent structures but secondarily involving thalamus and primary bilateral thalamic gliomas were excluded, but the contrallateral growth of a unilateral tumor was included in the study.

Clinical data, including age at presentation, sex, duration and type of symptoms, preoperative Karnofsky Performance Status (KPS), side, cystic appearance, maximal tumor diameter, treatment received, and status at the end of the follow-up period, were recorded. All patients underwent neuraxis magnetic resonance imaging (MRI) prior to treatment and during the follow-up period. All neuroimages and histological specimens were interpreted by two senior neuroradiologist and two neuropathologists, respectively. The T2/FLAIR images were used for defining the tumor diameter. And the enhancing lesion present was also considered together with the T2/FLAIR for defining tumor diameter.

### Treatment protocols

All included unilateral thalamus glioma (UTG) patients underwent tumor resection surgery aimed at achieving maximal safe tumor resection. The extent of tumor resection was determined on the basis of early postsurgical imaging and/or the neurosurgical report. Gross total tumor resection was defined as macroscopic total removal of the tumor mass, subtotal tumor resection was defined as a removal of ≥80 % of the tumor mass, and partial tumor resection was defined as <80 % resection. Various surgical approaches (the precentral interhemispheric transcallosal interforniceal approach, frontal transcortical approach, and parieto-occipital transventricular approach) were used. Postoperative adjuvant therapy was administrated based on the pathology diagnosis as well as the extent of tumor resection. All patients received prophylactic antiepileptic treatments postoperatively for 1 week.

### Statistical analysis

Statistical analysis was performed retrospectively using the SPSS statistical package 16.0 (SPSS Inc). Overall survival (OS) and progression-free survival (PFS) were determined using Kaplan-Meier analysis and a log-rank test. Events were defined as tumor relapse or progression, occurrence of a secondary malignancy, or death due to any cause. All of the clinical data of the one perioperative death and the other two lost cases (lost to follow-up after discharging from the hospital) were excluded prior to prognostic relevance analysis. All UTGs were defined according to the following parameters: sex, age at diagnosis, symptom lateral, cystic appearance of tumors, maximum tumor diameter, preoperative KPS status, grading of tumors, preoperative ventriculo-peritoneal (V-P) shunt, postoperative V-P shunt, and extent of tumor resection. The cutoff points were set as the median number for both categorical variables and continuous variables under consideration of the clinical practice (Table 5).

The prognostic relevance regarding PFS or OS of all remaining thalamic gliomas was compared to establish subgroups of the parameters defined above. The survival analysis was completed for the same subgroups using a Cox regression analysis. To compare the correlations between different subgroups, we performed the two-sided chi-square test to determine the significances for each parameter. The significance level was set at *P* < 0.05.

## Results

### Clinical, radiological features of patients with UTGs

Using the selection criteria, we identified 33 patients with UTGs. The clinical and histological characteristics of the 33 cases of UTGs in the present study are summarized in Table 1. Magnetic resonance images were available in all 33 UTGs (Table 2). The symptom duration from onset to hospital admission ranged from 1 week to 12 months; the median duration was 2 months. Among the 33 patients, 25 patients had headaches; 14 had motor deficits; 10 had hyperesthesia; 8 had blur vision; 7 had hypomnesia; 7 had dysphasia; and 1 had facial palsy. The median preoperative KPS was 60 (ranging from 40 to 80). The postoperative KPS was not collected.

**Table 1:** Clinical, radiological, and pathological features of 33 adult UTGs

No.	Age (years)	Sex	Tumor location	Side	Diameter (cm)	Resection	Pathology	Postop therapy	Follow-up time^a^	Status at the last follow-up
1	53	M	Th + Mid	Left	3.8	STR	A	Radio	25.5	Death with progression
2	20	F	Th + Mid	Right	4.1	PR	GBM	Radio	21	Death with progression
3	20	M	Th + IC	Right	4.2	STR	AA	Radio	17	Death with progression
4	33	M	Th + Mid	Right	4.1	GTR	OA	Radio	24	Death with progression
5	32	F	Th	Right	3.1	GTR	AA	Radio	24	Death with progression
6	47	F	Th + BG	Left	3.5	GTR	GBM	Radio	30	Death with progression
7	38	M	TH + Mid	Right	3.4	STR	AA	Radio	14	Death with progression
8	37	M	Th	Left	3.9	GTR	A	Radio	24	Death with progression
9	56	M	Th	Left	1.2	GTR	A	Radio	46	Alive without progression
10	42	M	Th	Right	3.2	GTR	GBM	Radio	49	Alive with progression
11	21	F	Th	Left	4.5	STR	AA	Radio	17	Death with progression
12	37	M	Th + IC	Left	3.5	GTR	GBM	Radio	14	Death with progression
13	29	M	Th + Mid + IC	Right	4.1	GTR	A	Radio	35	Death with progression
14	46	M	Th + IC	Left	5.3	GTR	GBM	–	1	Death without progression
15	33	M	Th + IC	Right	6.2	PR	GBM	–	–	Lost to follow-up
16	29	M	Th + IC + PiR	Right	4.5	GTR	OA^b^	Radio + chemo	25	Death with progression
17	49	F	Th	Right	3.5	GTR	AA	Radio	17	Death with progression
18	48	M	Th	Left	4.2	GTR	AO^b^	Radio + chemo	27	Death with progression
19	36	M	Th + IC + CTh	Right	3.2	PR	AOA^b^	Radio	20	Death without progression
20	24	M	Th + Mid + IC	Left	5.4	GTR	A^b^	–	14	Alive without progression
21	52	F	Th + CTh	Right	2.5	STR	AO^b^	Radio + chemo	2	Death with progression
22	21	F	Th + Mid	Right	3.8	STR	AOA^b^	Radio + chemo	12	Alive without progression
23	45	F	Th + Mid	Right	4.2	GTR	GBM^b^	Radio + chemo	3	Death without progression
24	30	M	Th	Right	3.9	GTR	GBM^b^	Radio + chemo	11	Alive with progression
25	43	M	Th	Right	4.7	GTR	AA^b^	Radio + chemo	11	Alive without progression
26	63	F	Th + IC	Left	4.1	GTR	OA^b^	Chemo	16	Alive with progression
27	47	F	Th + BG + IC	Left	3.4	PR	GBM^b^	Radio + chemo	4	Alive without progression
28	45	F	Th + IC	Right	4.8	PR	GBM^b^	–	3	Death without progression
29	20	M	Th + Mid	Right	5.3	STR	AA^b^	Radio + chemo	21	Death with progression
30	53	F	Th + CTh	Left	5.1	GTR	OA	–	–	Lost to follow-up
31	31	M	Th + Mid + IC	Left	5.2	STR	AOA^b^	Radio	24	Death with progression
32	66	M	Th + Mid + IC	Right	4.5	GTR	GSM	Chemo	1 week	Perioperative death
33	48	M	Th + Mid	Left	5.2	STR	GBM^b^	Radio + chemo	13	Death with progression

*Th* thalamus, *Mid* midbrain, *IC* internal capsule, *BG* basal ganglia, *PiR* pineal region, *CTh* contralateral thalamus, *GTR* gross total resection (≥90 % resection), *STR* subtotal resection (≥80 but <90 %), *PR* partial resection (<80 %), *A* astrocytoma, *AA* anaplastic astrocytoma, *AO* anaplastic oligodendroglioma, *AOA* anaplastic oligodendroastrocytoma, *GBM* glioblastoma, *GSM* gliosarcoma, *OA* oligoastrocytoma

^a^The follow-up time take the month as the unit

^b^The molecular pathologies were available

**Table 2:** Image features of 33 adult UTGs

Characteristics	No. (%) of patients
Location of tumors	
Confined in the thalamus	9 (27.3)
Extension to the brainstem	12 (36.4) (including 4 extensions to the basal ganglia or internal capsule and 1 extension to the contralateral thalamus)
Extension to the contralateral thalamus	3 (9.1) (including 1 extension to the brainstem and 1 extension to the internal capsule)
Extension to the basal ganglia and/or internal capsule	14 (42.4) (including 4 extensions to the brainstem, 1 extension to the contralateral thalamus, and 1 extension to the habenular commissure)
Cystic changes	
Yes	9 (27.3)
No	24 (72.7)
T1 and T2 signals	
Hypointense T1 and hyperintense T2	14 (42.4)
Hypointense T1 and mixed T2	2 (6.1)
Mixed T1 and hyperintense T2	11 (33.3)
Mixed T1 and T2	6 (18.2)
Enhancement	
Yes	30 (90.9)
No	3 (9.1)

All patients received maximal safe tumor resection under the guidance of intraoperative ultrasound monitoring. No patient received preoperative chemotherapy and/or radiotherapy. Most of the patients received postoperative adjuvant therapy (Table 1). Three patients received clinical follow-ups without further treatment. One of these patients was a middle-aged man who refused to receive and adjuvant therapy and died 3 months after gross total resection of the tumor due to glioblastoma, the second patient was a young man who remains alive with no progression of disease after receiving a gross total resection of his thalamic glioma with the diagnosis of astrocytoma, and the third patient was a middle-aged woman who refused to receive any adjuvant therapy and died 3 months after partial resection of the tumor due to glioblastoma.

### Perioperative management of cerebral spinal fluid problems

Ventriculomagely was identified in 27 of the 33 cases (81.8 %). Seven of the 27 cases (25.9 %) required an emergent V-P shunt. All 7 V-P shunts were performed in the ventricle contra-lateral to the lesion at 5 to 45 days (median 12 days) prior to tumor resection. The other 20 patients with some degree of ventriculomegaly were not treated, thereby leaving additional room to facilitate tumor resection through a transventricular approach.

After tumor resection, 4 patients with UTG (12.1 %) developed hydrocephalus and received V-P shunts in the ventricle contra-lateral to the lesion at 10, 13, 30, or 45 days after tumor resection, respectively.

### Surgical findings and outcomes of patients with UTGs

The surgical findings and outcomes are summarized in Table 3. The choice of the surgical approaches was based on the tumor location as well as the preferences of surgeon. The frontal transcortical approach was taken to remove tumors located in the anterior part of the unilateral thalamus in 6 cases, the precentral interhemispheric transcallosal interforniceal approach was taken to remove tumors located in the middle part of the unilateral thalamus in 5 cases, and the parieto-occipital transventricular approach was taken to remove tumors located in the posterior part of the unilateral thalamus in 22 cases. Of note, when the tumors are located in the posterior part of the left thalamus, the language function area should be cautiously protected. Perioperative death occurred in one case after tumor resection due to postoperative malignant brain edema.

**Table 3:** Treatment details of 33 adult UTGs

Characteristics	No. (%) of patients
Approaches	
Parieto-occipital transventriclular approach	22(66.7)
Frontal transcortical approach	6(18.2)
Precentral interhemispheric transcallosal interforniceal approach	5(15.2)
Extent of surgical resection	
Gross total tumor resection (≥90 %)	19 (57.6)
Subtotal tumor resection (≥80 but <90 %)	9 (27.3)
Partial tumor resection (<80 %)	5 (15.2)
Location of residual tumor	
Posterior part of the third ventricle	3 (20)^a^
Lateral part of the thalamus adjacent to the internal capsule	6 (40)^a^
Medial and internal part of the thalamus	6 (40)^a^
Preoperative ventricular peritoneal shunt	
Yes	7 (21.2)
No	26 (78.8)
Postoperative ventricular peritoneal shunt	
Yes	4 (12.1)
No	29 (87.9)
Postoperative adjuvant therapy	
Radiotherapy + chemotherapy	10 (30.3)
Radiotherapy	16 (48.5)
Chemotherapy	2 (6.1)

^a^Percentage refers to the comparison reasons of the total residual tumor numbers of the patients (100 %)

After tumor resection, headaches were significantly relieved or eliminated in 25 cases. Among the 14 patients with preoperative motor deficits, 4 cases demonstrated obvious improvement, 5 cases showed no significant changes, and 5 cases exhibited postoperatively deterioration, including the one death due to postoperative malignant brain edema. In the 10 cases with preoperative hyperesthesia, obvious improvement was observed in 2 cases; the other 8 cases showed no significant changes or deterioration. All 8 patients with blurred vision lost the symptom postoperatively. Among the 7 patients with hypomnesia, obvious improvement was observed in 5 cases and no significant improvement in the remaining 2. Among the 7 patients with dysphasia, obvious improvement was observed in 4 cases, no changes in 2 cases, and deterioration in 1 case. The one patient with facial palsy demonstrated no obvious improvements after operation and finally lost to follow-up.

After the operation, new hemiparesis occurred was observed in 8 cases. Transitory worsening of hemiparesis but significant improvement within 2 weeks was observed in 6 cases; no obvious improvement was observed in the other 2 cases at the last follow-up. New dysphasia was observed in 3 cases, with transitory worsening and spontaneously regression within 3 months in all 3 cases. No new occurrence of hyperesthesia was observed in this series. No patient had seizures requiring additional antiepileptic medication pre- or postoperatively.

### Recurrence and survival among patients with UTGs at follow-up

**Fig. 1:**
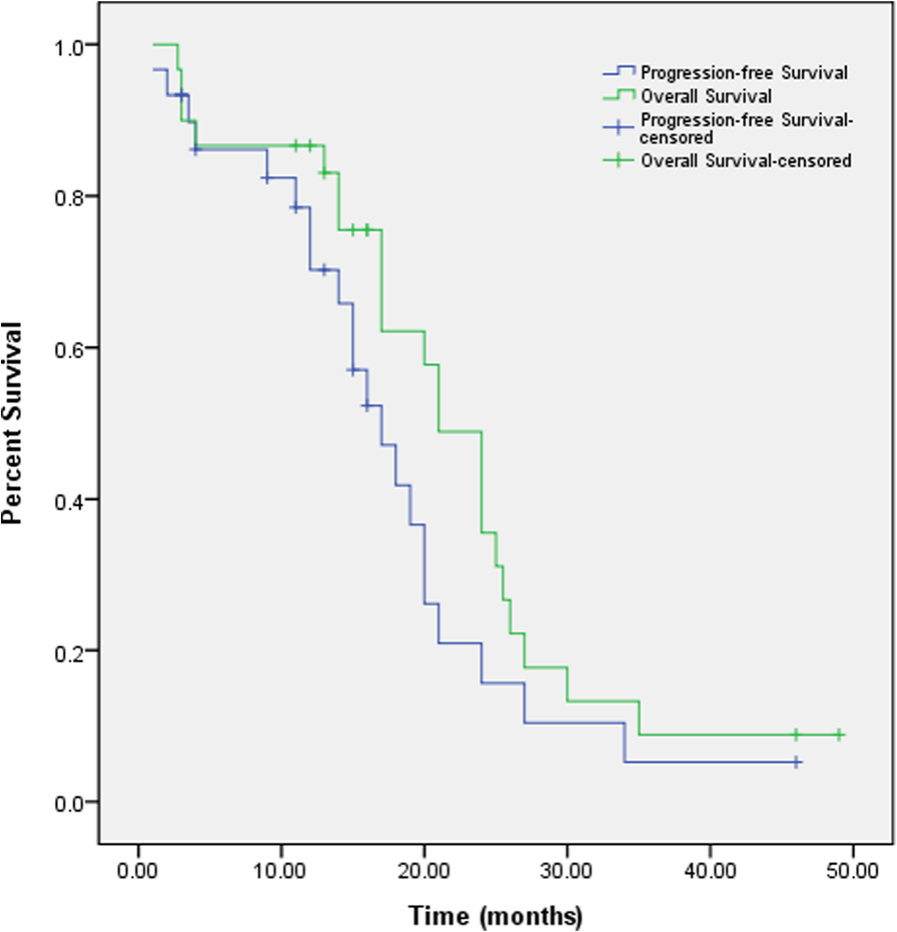
Kaplan-Meier plots of PFS and OS for 30 UTGs patients

The follow-up period ranged from 1 week to 49 months; the median follow-up time was 17 months. At the last follow-up, 21 patients had experienced tumor progression. The median PFS was not available. The 1-year and 2-year PFS rates were 51.5 and 9.1 %, respectively. Twenty-one patients died of recurrent tumors, and the median OS was not available. The 1-year and 2-year OS rates were 72.7 and 24.2 %, respectively. Kaplan-Meier plots of PFS and OS are shown in Fig. 1.

### Pathological features of UTGs

Pathological data are summarized in Table 1. Molecular pathology results were available in 15 of the 33 UTGs. Immunohistochemistrywas positive for P53 in 69.2 % of the cases (9/13), Ki-67 in 90.9 % (10/11), GFAP in 90.9 % (10/11), MGMT in 60 % (6/10), VEGF in 60 % (6/10), TOPO-II in 80 % (8/10), P-170 in 50 % (5/10), MMP-9 in 90 % (9/10), GST-π in 80 % (8/10), PTEN in 100 % (9/9), EGFR in 66.7 % (6/9), Oligo-2 in 100 % (9/9), etc. (Table 4).

**Table 4:** Molecular pathology features of 33 adult UTGs

Molecular pathologies		UTGs no. (%)	Molecular pathologies		UTGs no.(%)	Molecular pathologies		UTGs no. (%)
P53			TOPO-II			GST-π		
	+	9(69.2)		+	8(80)		+	8(80)
	−	4(30.7)		−	2(20)		−	2(20)
GFAP			P-170			PTEN		
	+	10(90.9)		+	5(50)		+	9(100)
	−	1(9.1)		−	5(50)		−	0(0)
MGMT			Oligo-2			EGFR		
	+	6(60)		+	9(100)		+	6(66.7)
	−	4(40)		−	0(0)		−	3(33.3)
VEGF			MMP-9					
	+	6(60)		+	9(90)			
	−	4(40)		−	1(10)			

### Clinical, radiological, and pathological parameters associated with PFS and OS

**Fig. 2:**
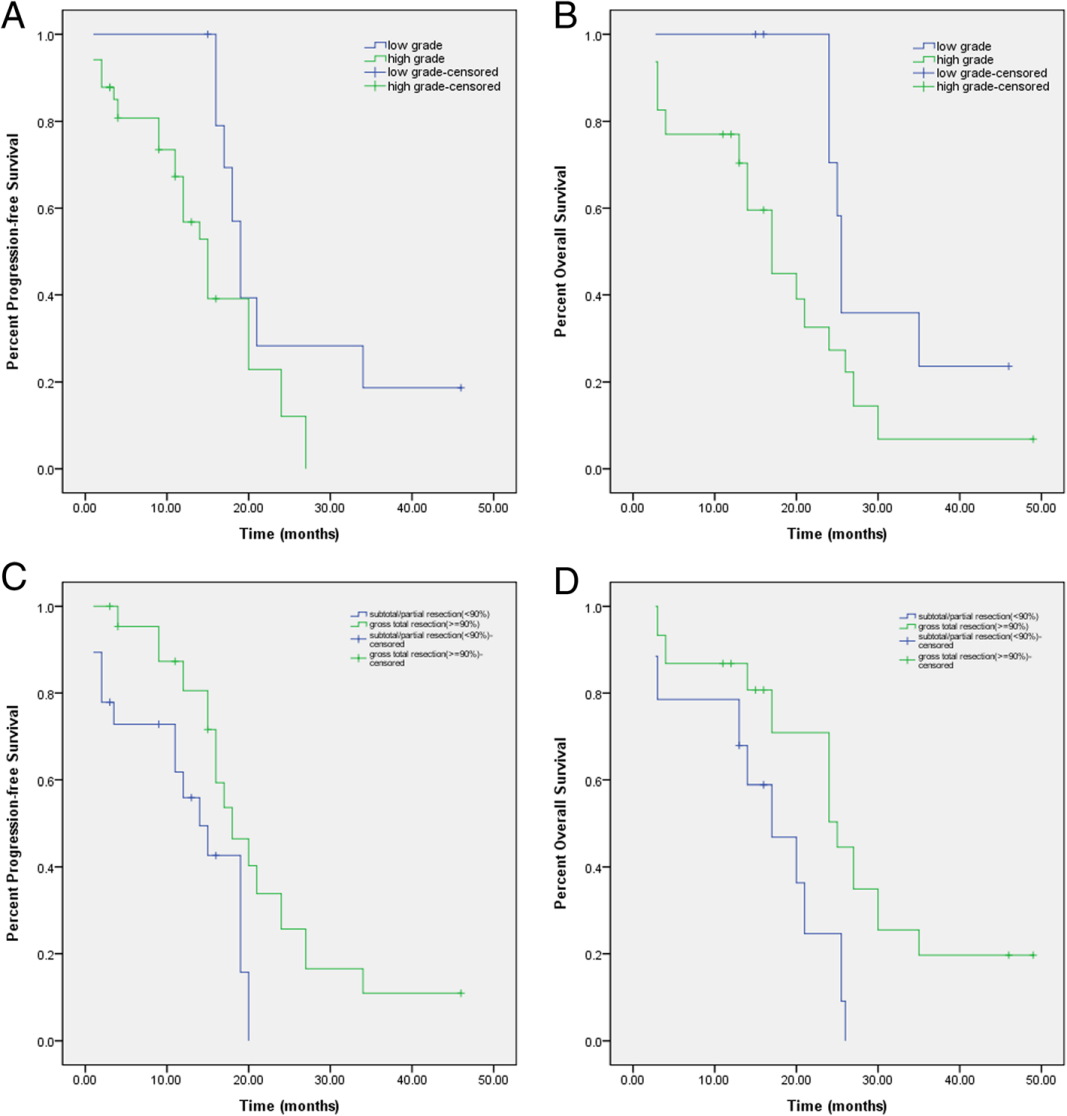
Patients with low-grade pathology have longer PFS (**a**) and OS (**b**) than patients with high-grade. Gross total tumor resection is significantly associated with longer PFS (**c**) and OS (**d**)

Parameters significantly associated with longer PFS and OS were determined using the log-rank test and Cox regression method (Table 5, Fig. 2, Additional files 1, 2, 3, 4, 5, 6, 7, and 8: Figures s1–s8). Univariate and multivariate analysis revealed several factors were correlated with a better PFS survival (Table 5). Also univariate and multivariate analysis revealed several factors were correlated with a better PFS survival (Table 5). Cox regression analysis revealed that age <42 years, female sex, symptom duration ≥2 months, cystic appearance, maximal tumor diameter <3.9 cm, preoperative KPS ≥60, and gross total resection were associated with a better PFS prognosis, while left side (*P* = 0.259) and lower-pathological grade (*P* = 0.531) failed to show statistical significance of a better PFS survival. Cox regression analysis revealed that age <42 years, female sex, left-sided tumor, symptom duration ≥2 months, cystic appearance, maximal tumor diameter <3.9 cm, lower-pathological grade, preoperative KPS ≥60, gross total resection, and postoperative V-P shunt were also associated with a better OS prognosis (Additional file 9: Tables S1 and S2).

**Table 5:** Parameters correlated with PFS and/or OS in 30 adult UTGs

Characteristics	Median PFS (INR)^a^	Progression-free survival rates (%)				*P* value	Median OS (INR)^a^	Overall survival rates (%)				*P* value
		6 months	12 months	18 months	24 months			6 months	12 months	18 months	24 months	
Age (years)												
<42	N/A	87.5	62.5	25.0	6.3	<0.001	N/A	100.0	93.8	56.3	18.8	<0.001
≥42	N/A	64.3	50.0	28.6	14.3		N/A	71.4	64.3	35.7	35.7	
Sex												
Male	N/A	78.9	47.4	31.6	10.5	0.037	N/A	94.7	84.2	57.9	36.8	<0.001
Female	N/A	72.7	72.7	18.2	9.1		N/A	72.7	72.7	27.3	9.1	
Lateral												
Left	N/A	84.6	69.2	30.8	15.4	<0.001	N/A	92.3	92.3	46.2	38.5	<0.001
Right	N/A	70.6	47.1	23.5	5.9		N/A	82.4	70.6	47.1	17.6	
Preoperative kps												
≥60	N/A	85.7	66.7	33.3	14.3	<0.001	N/A	95.2	90.5	52.3	23.8	<0.001
<60	9(3–20)	55.6	33.3	11.1	0.0		N/A	66.7	55.6	33.3	33.3	
Postoperative kps												
≥60	N/A	84.0	72.0	36.0	16.0	<0.001	N/A	92.0	84.0	52.0	40.0	<0.001
<60	N/A	40.0	20.0	0.0	0.0		N/A	60.0	60.0	20.0	20.0	
Symptom duration (months)												
≥2	N/A	93.3	73.3	40.0	20.0	<0.001	N/A	93.3	93.3	66.7	46.7	<0.001
<2	11(2–24)	60.0	40.0	13.3	0.0		N/A	80.0	66.7	26.7	6.7	
Cystic changes												
Yes	N/A	85.7	71.4	42.9	28.6	<0.001	N/A	100.0	100.0	85.7	57.1	<0.001
No	N/A	73.9	52.2	21.7	4.3		N/A	82.6	73.9	34.8	17.4	
Diameter (cm)												
<3.9	N/A	91.7	66.7	41.7	16.7	<0.001	N/A	91.7	91.7	50.0	33.3	<0.001
≥3.9	N/A	66.7	50.0	16.7	5.5		N/A	83.3	72.2	44.4	22.2	
Pathology												
Low grade	N/A	100.0	100.0	50.0	25.0	<0.001	N/A	100.0	100.0	75.0	50.0	<0.001
High grade	N/A	68.2	40.9	18.2	4.5		N/A	81.8	72.7	36.4	18.2	
Postoperative v-p shunt												
Yes	14.5(12–24)	100.0	75.0	25.0	0.0	–	N/A	100.0	100.0	50.0	25.0	<0.001
No	N/A	73.1	53.8	26.9	11.5		N/A	84.6	76.9	46.2	26.9	
Resection												
Gtr	N/A	82.4	64.7	35.3	17.6	<0.001	N/A	88.2	76.5	52.9	35.3	<0.001
Str/pr	12(2–20)	69.2	46.2	15.4	0.0		N/A	84.6	84.6	38.5	15.4	

*N/A* not available

^a^Median PFS and median OS was calculated based on the final status of follow-up

^b^Means no correlation in univariate analysis

### Illustrative cases

#### Case 1

**Fig. 3:**
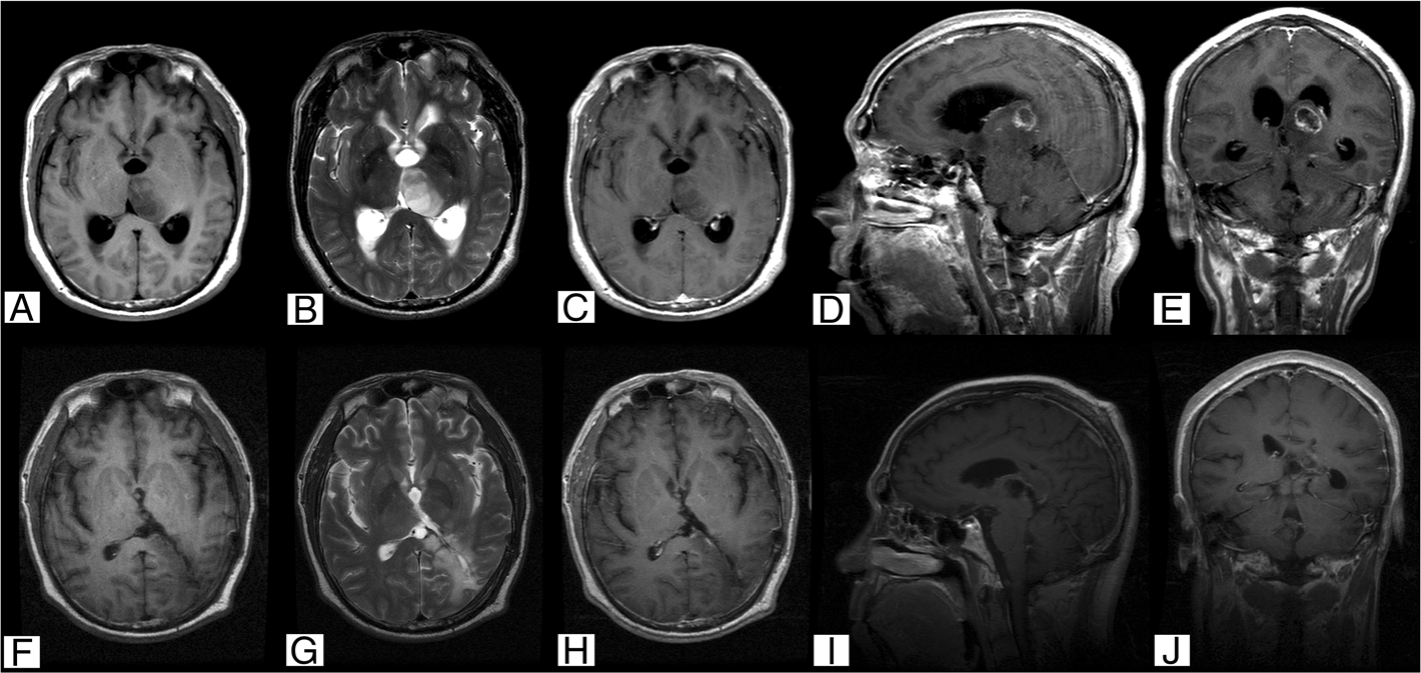
Case 1. Cranial MRI examination revealed unilateral thalamus glioma located in the left thalamus and midbrain with hypointense T1 (**a**) and hyperintense T2 (**b**) signals, which were heterogeneously enhanced after injecting contrast agent (**c**–**e**). Postoperative MRI confirmed subtotal resection (**f**–**j**). Pathological examination revealed a diagnosis of astrocytoma (WHO Grade II). Original magnification ×100

A lesion affecting the left thalamus and midbrain was diagnosed radiologically in a 53-year-old male admitted to our hospital. He reported having headache for the past year and hypomnesia for the past 6 months. Neurological examination revealed no obvious abnormal findings. Radiological examination revealed a lesion located in the left thalamus and affecting the midbrain with hypointense T1 and hyperintense T2 signals as well as partial heterogeneous enhancement (Fig. 3). Thalamic glioma was diagnosed, and the patient underwent a subtotal resection of the tumor using a parieto-occipital transventricular approach. During surgery, the tumor appeared as a pinkish-gray soft mass with a poorly defined border and a cystic component of dark-yellow fluid in the posterior part of the mass. The postoperative pathology diagnosis was astrocytoma (WHO grade II). No immunohistochemical staining was performed. The patient suffered from a transient deterioration of the right limb motor function and language nonfluency after surgery. After 2 weeks, his limb movements returned to normal and his language fluency recovered. The patient was discharged 23 days after operation. Postoperative cranial radiotherapy was performed. However, the tumor progressed after 19 months. The patient refused to undergo another surgery, chemotherapy, or radiotherapy and died 25.5 months after the operation.

#### Case 33

**Fig. 4:**
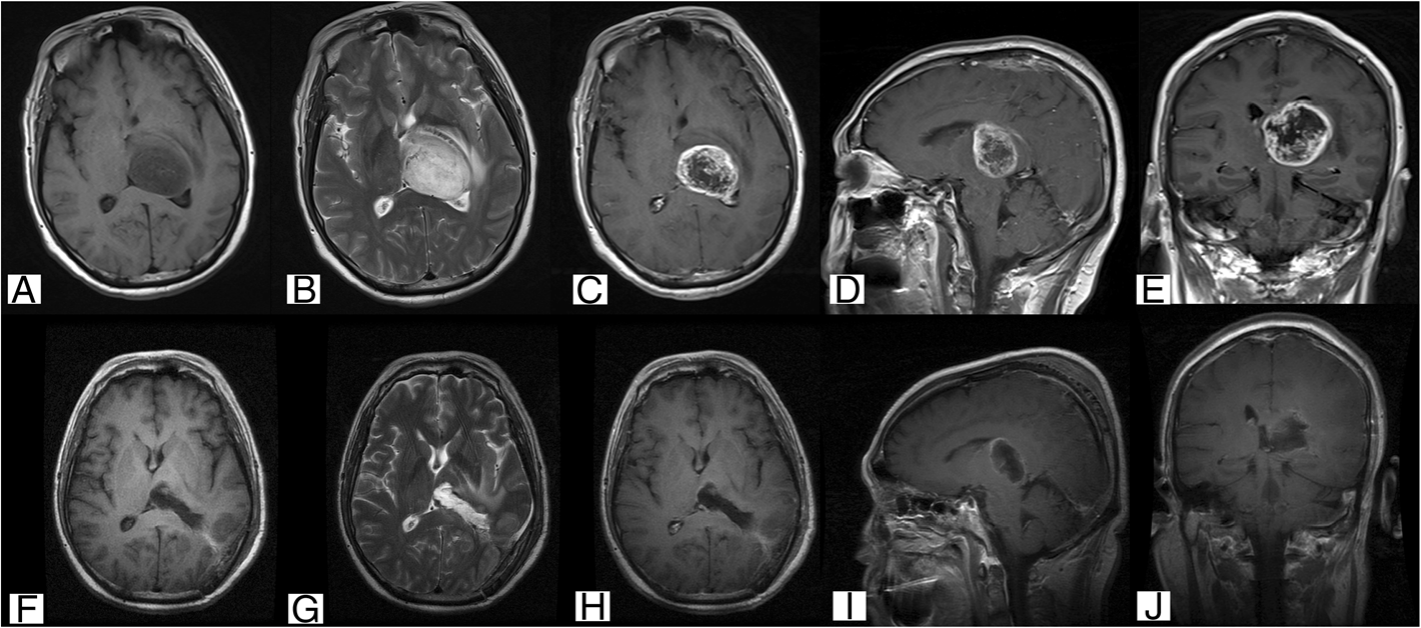
Case 33. Cranial MRI examination revealed unilateral thalamus glioma located in the right thalamus and midbrain with mixed hypointense and hyperintense (**a**) and hyperintense T2 (**b**) signals with clear enhancement (**c**–**e**). Postoperative MRI confirmed subtotal resection (**f**–**j**). Original magnification ×100

A pathologic diagnosis of anaplastic astrocytoma with transformation to glioblastoma was confirmed in a 48-year-old male admitted to our hospital. He complained of a motor dysfunction of his right lower limb for the past 2 weeks. Neurological examination revealed weakness of his right lower limbs. The lesion showed mixed hypointense and isointense T1 and hyperintense T2 signals with clear enhancement (Fig. 4). The patient underwent a gross total resection using a parieto-occipital transventricular approach. During the operation, the tumor was found to be a gray-yellow soft mass with a poorly defined border. The postoperative pathology report confirmed the diagnosis of anaplastic astrocytoma with transformation to glioblastoma, which was immunopositive for MGMT, PTEN, GFAP, Oligo-2, MMP-9, P53 and immunonegative for EGFR, P-170, VEGF, TOPO-II, and GST-π. Limb weakness partially improved over a span of 2 weeks, and the patient was discharged 15 days after surgery. The postoperative follow-up revealed tumor recurrence at 1.5 months after surgery. The patient underwent postoperative concurrent chemoradiotherapy (radiotherapy + temozolomide) plus 10 cycles of temozolomide chemotherapy. The patient died 13 months after surgery.

## Discussion

Several studies on thalamic glioma have been reported; however, the patient outcomes have been poor regardless of the type of treatment, and no comprehensive guidelines with regard to treatment are available [4–7, 11, 30]. Recent studies have demonstrated improved survival of pediatric patients with thalamic glioma after an aggressive tumor resection followed by postoperative adjuvant therapy [15, 28, 29]. However, the prognosis of thalamic glioma in adult patients after aggressive surgical treatment remains uncertain because adult thalamic glioma cases have always been assessed together with those of pediatric patients [3, 11, 18, 30–32], and no studies have been devoted solely to adult thalamic glioma. Therefore, the present retrospective study was undertaken to investigate the clinical features of unilateral thalamic gliomas in adults as well as to analyze the parameters that are likely to influence survival.

### Basic clinical features correlated with prognosis

In the current series, several basic clinical features were found to correlate with a better prognosis, namely, age <42 years, symptom duration ≥2 months, and preoperative KPS ≥60. Some of the results in our series are in accordance with previously reported studies.

Age <42 years correlated with longer survival. This age of onset result was consistent with those of previous studies [1, 5, 33]. Moreover, previous studies also revealed better outcome in thalamic tumor patients who were younger than 40 years compared with older patients [5]. Similarly, Nishio et al. also demonstrated that the outcome of younger patients was better than that of older patients despite the same pathological diagnosis [4].

A symptom duration of ≥2 months was associated with longer survival, a finding in agreement with a previous study [15]. Moreover, in our study, we found that a short duration of symptoms is often indicative of a high-grade glioma (WHO grades III and IV). In the subgroup of patients with symptom durations <2 months, the rate of high-grade glioma was 100 % (compared with a rate of 46.7 % in patients with symptom durations ≥2 months) (*P* = 0.002). This result is similar to the reports of Cuccia [9].

A preoperative KPS ≥60 was correlated with longer survival and may be an independent factor for better OS. This result may reflect the fact that lower KPS may result in a decreased ability of patients to withstand neurological insults caused by the tumor, surgery, and/or adjuvant therapies [34–36]. The median KPS of the current study was 60; a lower KPS score was observed in 9 cases (30 %), which is substantially lower rate than previously reported [6, 37]. The result of this study indicates that performance of thalamic patients was substantially worse on admission than for patients with glioma in other parts of the brain, which points to a higher surgical risk when performing a tumor resection in these patients. Of note, the 3 (preoperative KPS categories 40, 50, and 60, respectively) developed within 2 weeks after surgery in this group of patients.

### Tumor biological features correlated with prognosis

Tumors with cystic appearance were correlated with longer survival. The rate of cystic appearance in our study was 24.2 %, which is similar to the rate of 11.5–53 % in previous studies [13, 15, 38]. All cystic components as well as the walls of the cysts were completely excised during surgery. This approach is in accordance with Moshel’s study because cystic thalamic tumors also contain tumor cells in the cyst wall [38]. The pathology of tumors with cystic appearance in our study included astrocytoma in 4 cases, oligodendroastrocytoma in 1 case, anaplastic astrocytoma in 1 case, anaplastic oligodendroastrocytoma in 1 case, and glioblastoma in 1 case. No thalamus pilocytic astrocyomas were present in our study; this result is notably different from the thalamic tumors observed in children [15, 39].

A maximal tumor diameter of <3.9 cm correlated with longer survival. Few studies have indicated a correlation between tumor diameter and survival [11, 16]. Although, a previous study on pediatric thalamic glioma, Kramm failed to show a significant correlation between tumor diameter and survival [16], larger tumors may present more challenges to surgeons [38].

Low-grade pathology was correlated with longer survival and was also an independent factor for better OS. Tumor pathology has been generally accepted as key factor influencing survival [8, 28, 33]. Modern neuroimaging methods have contributed to a better understanding of the patterns of tumor growth. Thalamic gliomas are thought to grow along three primary pathways: along the white matter, underneath the ventricular ependyma, and through the cerebralspinal fluid pathways [8, 40]. Subsequently, surrounding tissues, such as the internal capsule, the basal ganglia, the mesencephalic tegmentum, and the hypothalamic structures (and their corresponding functions), may be affected [22, 41]. From this point of view, patients with glioma of higher pathology grade may be more strongly affected by the malignancy features and face a higher risk during surgery and other comprehensive treatments.

### Clinical treatment correlated with prognosis

CSF has long been a problem in thalamic glioma patients. Thalamic gliomas are apt to cause obstruction of the third or lateral ventricles. V-P shunt is one of the most accepted procedures for maintaining CSF circulation. Baroncini discussed CSF obstruction in pediatric thalamic glioma patients [28]; however, the correlation between V-P shunt and patient survival has never been examined. In the present study, we analyzed the correlation between survival and V-P shunt due to perioperative acute hydrocephalus. We found that postoperative V-P shunt correlated with longer OS. The infiltrative nature and the growth pattern of thalamic glioma likely contribute to the worsening of patients’ clinical status [40]. Based on these results, we also hypothesize that the occlusion of CSF circulation may be another main contributor to the poor prognosis because ventriculomagely and/or hydrocephalus were present in nearly all reported series.

Gross total tumor resection correlated with longer survival. Thalamic gliomas have long been a challenge and are considered difficult to excise. Most of these tumors received only partial resection, biopsy, or only radiotherapy for palliative care in earlier studies. However, with the advancement of microsurgical techniques, recent studies have reported improved survival following aggressive tumor resection in pediatric thalamic glioma patients [15, 28, 29], which indicates that current cases of thalamic glioma can be resected with acceptable complications nowadays. The present study, focused exclusively on adult UTG patients, found a statistically significant correlation between gross total tumor resection and survival. Therefore, more aggressive cytoreductive treatments should also be considered for these tumors.

### Study limitations

This retrospective study was based on a small series of cases. Consequently, our conclusions were based on the statistical analysis of a small number of patients and should be interpreted with caution.

## Conclusions

Unilateral thalamic glioma in adults represents a distinct clinical subtype of thalamic gliomas. Tumors with specific features, such as cystic appearances in radiology, may indicate better prognosis. Moreover, using perioperative ultrasound techniques and improved surgical techniques, maximal safe resection was often feasible and provided prognostic benefits with UTGs. However, the more fundamental biological features and the recommendation of maximal safe resection for UTGs whenever possible to promote prolonged survival still need to be further investigated in larger series.

## Additional files

Additional file 1: Figure S1. Age <42 years is significantly associated with longer PFS (A) and OS than age ≥42 years (B). (TIF 55 kb) Additional file 2: Figure S2. Male patients have longer PFS (A) and OS (B) than female patients. (TIF 56 kb) Additional file 3: Figure S3. Patients with left-sided tumor have a longer PFS (A) and OS (B) than patients with right-sided tumor. (TIF 58 kb) Additional file 4: Figure S4. Patients with preoperative KPS ≥60 have longer PFS (A) and OS (B). (TIF 57 kb) Additional file 5: Figure S5. Patients with symptom duration longer than 2 months have longer PFS (A) and OS (B). (TIF 57 kb) Additional file 6: Figure S6. Patients with cystic changes of tumor is significantly associated with longer PFS (A) and OS than patients without this changes (B). (TIF 55 kb) Additional file 7: Figure S7. Maximal tumor diameter <3.9 cm is associated with longer PFS (A) and OS (B). (TIF 56 kb) Additional file 8: Figure S8. Postoperative V-P shunt is significantly associated with longer OS. (TIF 40 kb) Additional file 9: Tables s1 and s2. Cox-regression analysis for PFS and OS, respectively. (DOCX 41 kb)
