# Supplementary figures and images for: Clinical, radiological, and pathological features of 33 adult unilateral thalamic gliomas
Source: World J Surg Oncol. 2016 Mar 10;14:78. doi: 10.1186/s12957-016-0820-x (PMC4785741; doi:10.1186/s12957-016-0820-x)

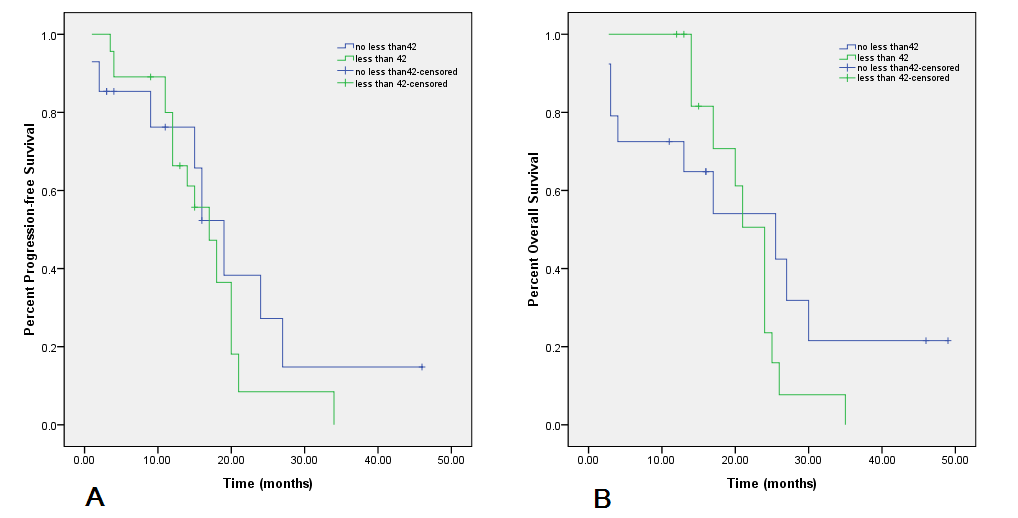

Supplement: Additional file 1: Figure S1. — Age <42 years is significantly associated with longer PFS (A) and OS than age ≥42 years (B). (TIF 55 kb) [file 12957_2016_820_MOESM1_ESM.tif]

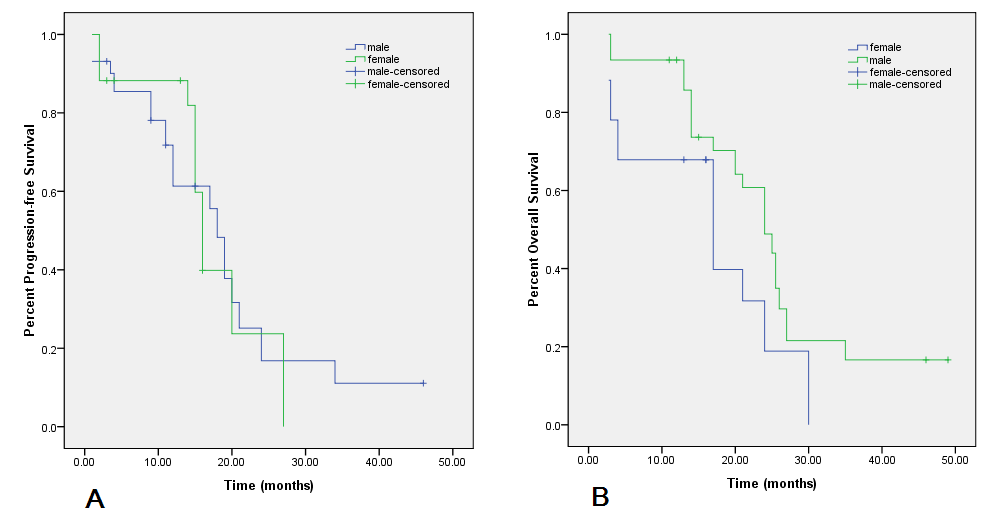

Supplement: Additional file 2: Figure S2. — Male patients have longer PFS (A) and OS (B) than female patients. (TIF 56 kb) [file 12957_2016_820_MOESM2_ESM.tif]

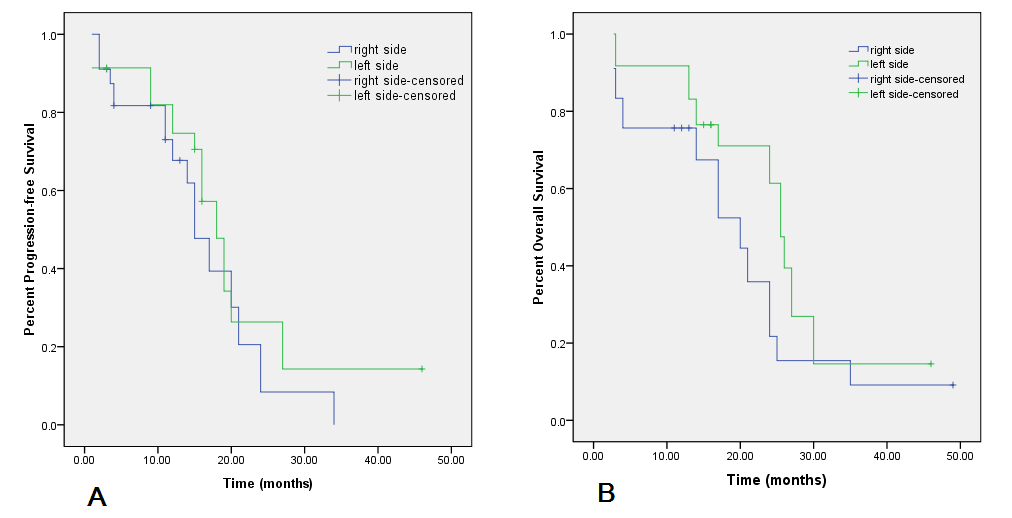

Supplement: Additional file 3: Figure S3. — Patients with left-sided tumor have a longer PFS (A) and OS (B) than patients with right-sided tumor. (TIF 58 kb) [file 12957_2016_820_MOESM3_ESM.tif]

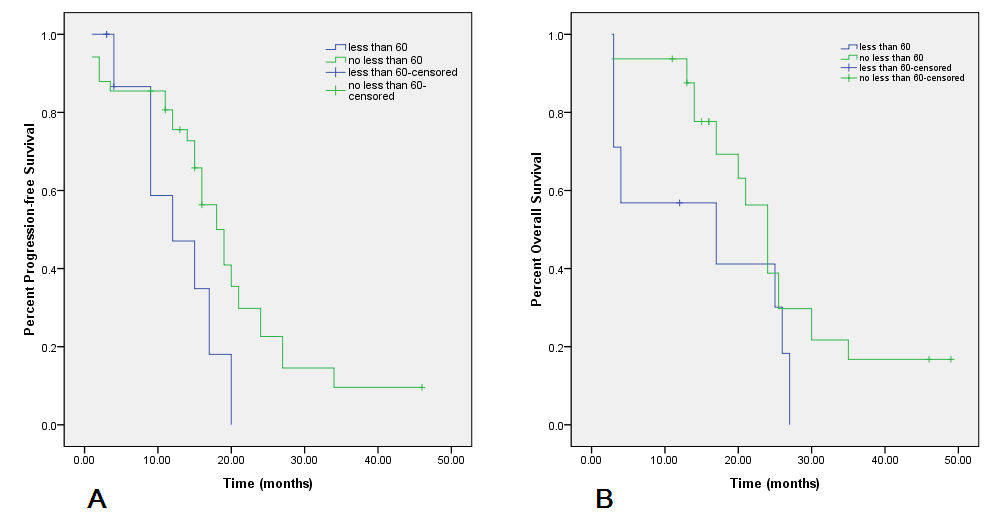

Supplement: Additional file 4: Figure S4. — Patients with preoperative KPS ≥60 have longer PFS (A) and OS (B). (TIF 57 kb) [file 12957_2016_820_MOESM4_ESM.tif]

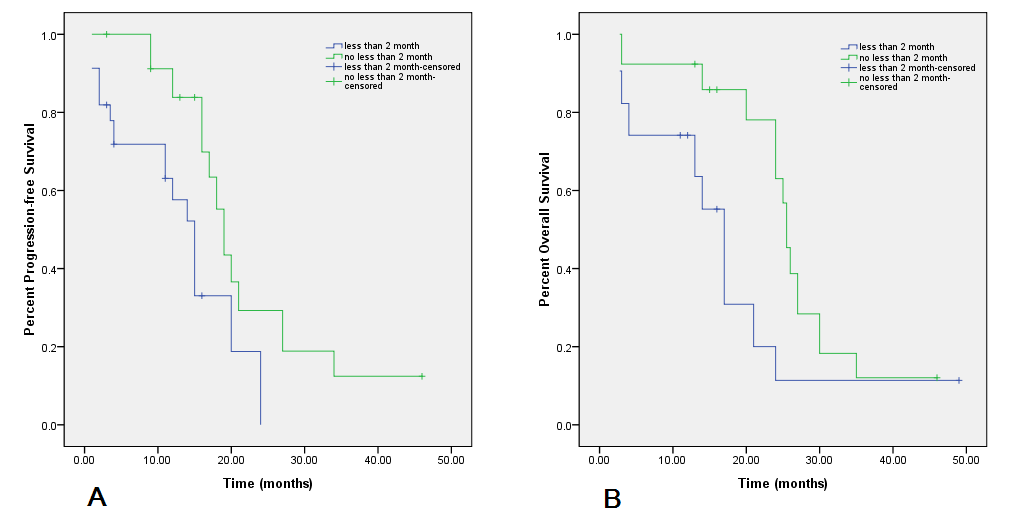

Supplement: Additional file 5: Figure S5. — Patients with symptom duration longer than 2 months have longer PFS (A) and OS (B). (TIF 57 kb) [file 12957_2016_820_MOESM5_ESM.tif]

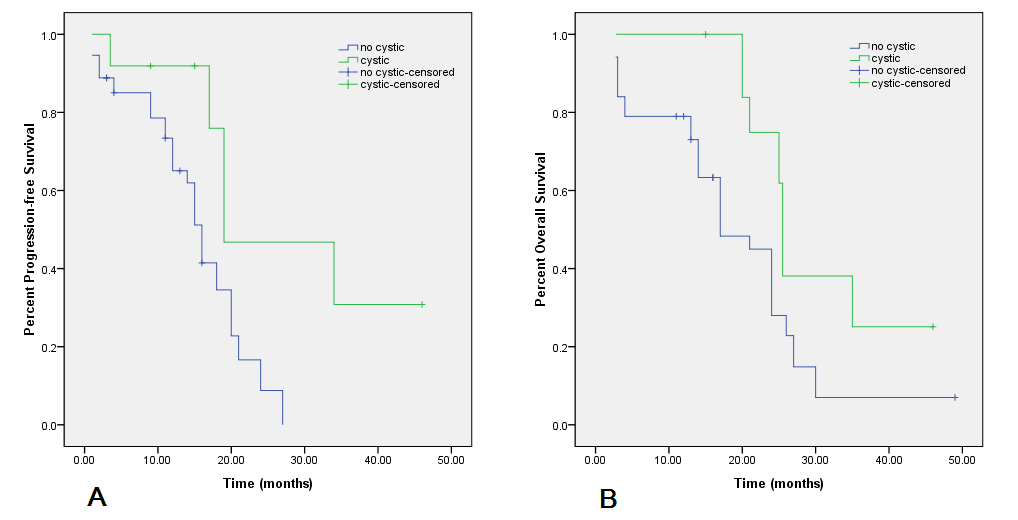

Supplement: Additional file 6: Figure S6. — Patients with cystic changes of tumor is significantly associated with longer PFS (A) and OS than patients without this changes (B). (TIF 55 kb) [file 12957_2016_820_MOESM6_ESM.tif]

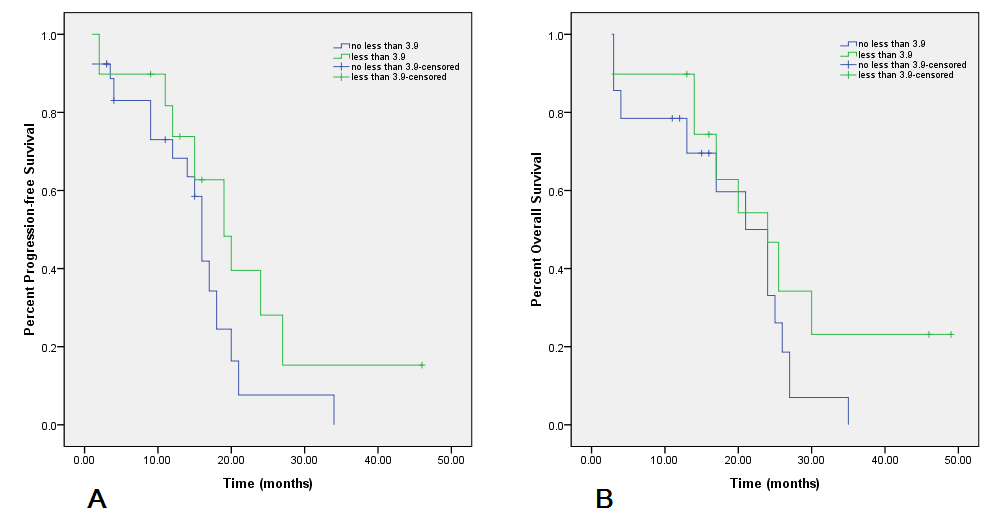

Supplement: Additional file 7: Figure S7. — Maximal tumor diameter <3.9 cm is associated with longer PFS (A) and OS (B). (TIF 56 kb) [file 12957_2016_820_MOESM7_ESM.tif]

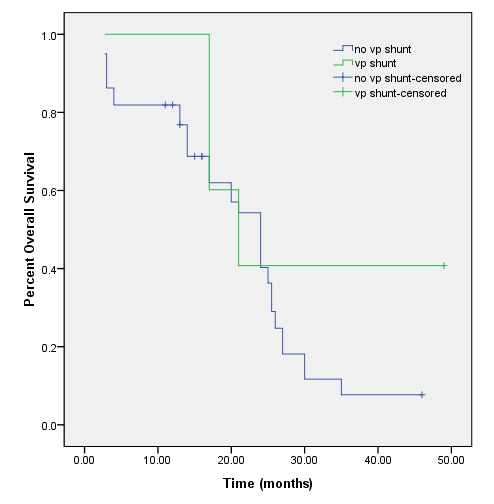

Supplement: Additional file 8: Figure S8. — Postoperative V-P shunt is significantly associated with longer OS. (TIF 40 kb) [file 12957_2016_820_MOESM8_ESM.tif]
