# Supplementary material for: Clinical, radiological, and pathological features of 33 adult unilateral thalamic gliomas
Source: World J Surg Oncol. 2016 Mar 10;14:78. doi: 10.1186/s12957-016-0820-x (PMC4785741; doi:10.1186/s12957-016-0820-x)

Table s1 Cox-Regression Analysis for PFS

| **Omnibus Tests of Model Coefficients^a,b^** | | | | | | | | | | | | | | | | | | | | | | |
| --- | --- | --- | --- | --- | --- | --- | --- | --- | --- | --- | --- | --- | --- | --- | --- | --- | --- | --- | --- | --- | --- | --- |
| Step | | -2 Log Likelihood | | | | Overall (score) | | | | | | | | | | Change From Previous Block | | | | | | |
|  |  |  |  |  |  | Chi-square | | | df | | | | Sig. | | | Chi-square | | df | | | Sig. | |
| 7 | | 9028.262 | | | | 491.008 | | | 7 | | | | .000 | | | 493.463 | | 7 | | | .000 | |
| a. Beginning Block Number 0, initial Log Likelihood function: -2 Log likelihood: 9521.725 | | | | | | | | | | | | | | | | | | | | | | |
| b. Beginning Block Number 1. Method = Forward Stepwise (Likelihood Ratio) | | | | | | | | | | | | | | | | | | | | |  | |
| **Variables in the Equation** | | | | | | | | | | | | | | | | | | | | | | |
|  | |  | | | B | | | SE | | | Wald | | df | | | Sig. | | Exp(B) | | 95.0% CI for Exp(B) | | |
|  | |  | | |  |  |  |  |  |  |  |  |  |  |  |  |  |  |  | Lower | Upper | |
| Step 7 | | sex | | | -.232 | | | .091 | | | 6.480 | | 1 | | | .011 | | .793 | | .663 | .948 | |
|  |  | cystic | | | -1.043 | | | .130 | | | 63.930 | | 1 | | | .000 | | .352 | | .273 | .455 | |
|  |  | agegroup | | | -.323 | | | .101 | | | 10.155 | | 1 | | | .001 | | .724 | | .594 | .883 | |
|  |  | durationgroup | | | -.940 | | | .095 | | | 97.470 | | 1 | | | .000 | | .390 | | .324 | .471 | |
|  |  | KPSgroup | | | .353 | | | .119 | | | 8.844 | | 1 | | | .003 | | 1.423 | | 1.128 | 1.795 | |
|  |  | Diametergroup | | | -1.207 | | | .115 | | | 110.361 | | 1 | | | .000 | | .299 | | .239 | .375 | |
|  |  | resection | | | -1.042 | | | .099 | | | 109.681 | | 1 | | | .000 | | .353 | | .290 | .429 | |
| **Variables not in the Equation^a^** | | | | | | | | | | | | | |  |  |  |  |  |  |  |  |  |
|  | | |  | | Score | | | df | | | Sig. | | |  |  |  |  |  |  |  |  |  |
| Step 7 | | | lateral | | 1.272 | | | 1 | | | .259 | | |  |  |  |  |  |  |  |  |  |
|  |  |  | pathology2 | | .392 | | | 1 | | | .531 | | |  |  |  |  |  |  |  |  |  |
| a. Residual Chi Square = 1.477 with 2 df Sig. = .478 | | | | | | | | | | | | | |  |  |  |  |  |  |  |  |  |


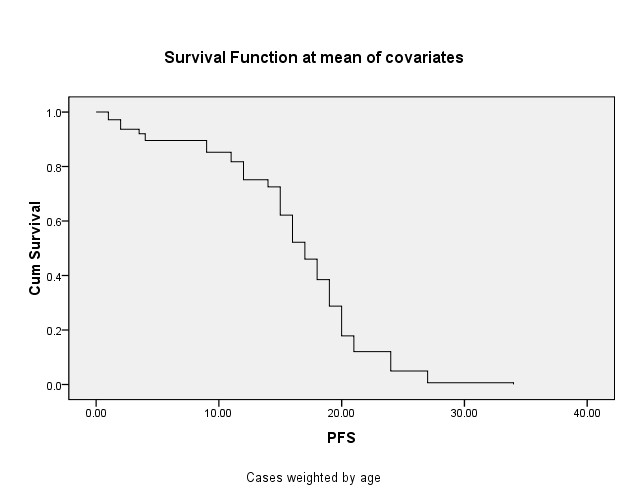


Table s2 Cox-Regression Analysis for OS

| **Omnibus Tests of Model Coefficients^a,b^** | | | | | | | | | | | | | | | | | |
| --- | --- | --- | --- | --- | --- | --- | --- | --- | --- | --- | --- | --- | --- | --- | --- | --- | --- |
| Step | | -2 Log Likelihood | | Overall (score) | | | | | | | Change From Previous Block | | | | | | |
|  |  |  |  | Chi-square | | df | | | Sig. | | Chi-square | | df | | | Sig. | |
| 10 | | 9184.779 | | 891.987 | | 10 | | | .000 | | 1004.761 | | 10 | | | .000 | |
| a. Beginning Block Number 0, initial Log Likelihood function: -2 Log likelihood: 10189.540 | | | | | | | | | | | | | | | | | |
| b. Beginning Block Number 1. Method = Forward Stepwise (Likelihood Ratio) | | | | | | | | | | | | | | | |  | |
| **Variables in the Equation** | | | | | | | | | | | | | | | | | |
|  | |  | | B | | SE | | Wald | df | | Sig. | | Exp(B) | | 95.0% CI for Exp(B) | | |
|  | |  | |  |  |  |  |  |  |  |  |  |  |  | Lower | Upper | |
| Step 10 | | sex | | -.612 | | .103 | | 35.500 | 1 | | .000 | | .542 | | .443 | .663 | |
|  |  | lateral | | -1.452 | | .116 | | 157.669 | 1 | | .000 | | .234 | | .187 | .294 | |
|  |  | cystic | | -1.678 | | .151 | | 123.518 | 1 | | .000 | | .187 | | .139 | .251 | |
|  |  | agegroup | | -.795 | | .108 | | 54.424 | 1 | | .000 | | .452 | | .366 | .558 | |
|  |  | durationgroup | | -1.894 | | .153 | | 153.694 | 1 | | .000 | | .150 | | .112 | .203 | |
|  |  | KPSgroup | | -.956 | | .133 | | 51.888 | 1 | | .000 | | .384 | | .296 | .498 | |
|  |  | Diametergroup | | -.742 | | .112 | | 43.513 | 1 | | .000 | | .476 | | .382 | .594 | |
|  |  | pathology2 | | -1.310 | | .185 | | 50.087 | 1 | | .000 | | .270 | | .188 | .388 | |
|  |  | postopvpshunt | | -3.148 | | .176 | | 321.635 | 1 | | .000 | | .043 | | .030 | .061 | |
|  |  | resection | | -2.217 | | .116 | | 363.772 | 1 | | .000 | | .109 | | .087 | .137 | |


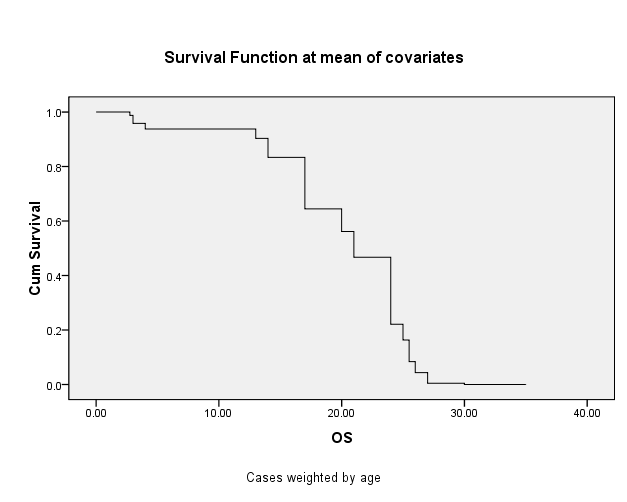

Supplement: Additional file 9: — Tables s1 and s2. Cox-regression analysis for PFS and OS, respectively. (DOCX 41 kb) [file 12957_2016_820_MOESM9_ESM.docx]
